# Assessing knowledge, attitudes, and practice of health providers towards the provision of postpartum intrauterine devices in Nepal: a two-year follow-up

**DOI:** 10.1186/s12978-021-01099-7

**Published:** 2021-02-17

**Authors:** Lucy Stone, Mahesh C. Puri, Muqi Guo, Iqbal H. Shah

**Affiliations:** 1 Independent Consultant, Kathmandu, Nepal; 2 Center for Research On Environment, Health and Population Activities (CREHPA), Kusunti, Lalitpur, P.O. Box 9626, Kathmandu, Nepal; 3 Harvard T. H. Chan School of Public Health, 665 Huntington Avenue, Boston, MA 02115 USA

**Keywords:** Health providers, Knowledge, Attitudes, Practice, KAP, Postpartum IUD, Postpartum family planning, Nepal

## Abstract

**Background:**

Health service providers play a key role in addressing women’s need for postpartum pregnancy prevention. Yet, in Nepal, little is known about providers’ knowledge, attitudes, and practice (KAP) on providing postpartum family planning (PPFP), particularly the immediate postpartum intrauterine device (PPIUD). This paper assesses providers KAP towards the provision of PPIUDs in Nepal prior to a PPIUD intervention to gain a baseline insight and analyzes whether their KAP changes both 6 and 24 months after the start of the intervention.

**Methods:**

Data come from a randomized trial assessing the impact of a PPIUD intervention in Nepal between 2015 and 2017. We interviewed 96 providers working in six study hospitals who completed a baseline interview and follow-up interviews at 6 and 24 months. We used descriptive analysis, McNemar’s test and the Wilcoxon signed-rank test to assess KAP of providers over 2 years.

**Results:**

The PPIUD KAP scores improved significantly between the baseline and 6-month follow-up. Knowledge scores increased from 2.9 out of 4 to 3.5, attitude scores increased from 4 out of 7 to 5.3, and practice scores increased from 0.9 out of 3 to 2.8. There was a significant increase in positive attitude and practice between 6 and 24 months. Knowledge on a women’s chance of getting pregnant while using an IUD was poor. Attitudes on recommending a PPIUD to different women significantly improved, however, attitudes towards recommending a PPIUD to unmarried women and women who have had an ectopic pregnancy improved the least. Practice of PPIUD counseling and insertion improved significantly from baseline to 24 months, from 10.4 and 9.4% to 99% respectively.

**Conclusions:**

Although KAP improved significantly among providers during the PPIUD intervention, providers’ knowledge on a women’s chance of getting pregnant while using an IUD and attitudes towards recommending a PPIUD to unmarried women and women who have had an ectopic pregnancy improved the least. Provider KAP could be improved further through ongoing and more in-depth training to maintain providers’ knowledge, reduce provider bias and misconceptions about PPIUD eligibility, and to ensure providers understand the importance of birth spacing.

## Plain english summary

In low-and middle-income countries, birth to pregnancy intervals that are less than 24 months are a risk to the health and lives of many women and children. Effective postpartum family planning (PPFP) including the immediate postpartum intrauterine device (PPIUD) offers women the opportunity to effectively space their births, significantly reducing maternal and child mortality and morbidity. Health providers play a vital role in providing knowledge, information, and access to PPFP to women who want to prevent postpartum pregnancies, however, in Nepal little is known about providers’ knowledge, attitudes, and practice (KAP) towards providing PPFP and the PPIUD. Using data from a PPIUD intervention in Nepal between 2015 and 2017, this study aims to fill this gap by assessing providers’ KAP towards PPIUD provision before the PPIUD intervention and analyze whether it changes 6 and 24 months after the start of the intervention. Significant improvements in provider KAP were found between the baseline and 6 months and additional improvements to attitudes and practice were found between 6 and 24 months into the intervention. Nonetheless, there were poor attitudes among providers towards recommending a PPIUD to unmarried women and women who have previously had an ectopic pregnancy. Throughout the intervention, providers also had persistently dim views on the importance of protecting women from pregnancy during their first postpartum year. To mitigate this, continued and more in-depth training is required to maintain providers’ knowledge, reduce provider bias and misconceptions about PPIUD eligibility, and to ensure providers understand the importance of postpartum birth spacing.

## Background

Untimely and unintended pregnancies in low-and middle-income countries (LMICs) continue to be a public health concern due to increased risks of negative maternal, neonatal and child pregnancy outcomes [1–3]. The World Health Organization (WHO) advises that women have a 2 year birth to pregnancy interval to reduce the risks of negative health outcomes. However, in some LMICs the birth to pregnancy interval in 50% or more pregnancies was less than 24 months [4, 5]. Postpartum family planning (PPFP) offers women the opportunity to effectively space their births, significantly reducing maternal and child mortality and morbidity [6, 7]. Despite this, 21% of births in Nepal occurred less than 2 years postpartum and over half of women less than 2 years postpartum have an unmet need for family planning [8, 9].

Long-acting reversible contraceptive (LARC) methods, such as the intrauterine device (IUD) is proven to be an effective, safe, reliable, and long-acting (up to 12 years) method of family planning that prevents unintended pregnancy and results in optimal birth spacing [10–12]. Half of health facilities in Nepal claim to supply LARCs including the IUD, however, in reality only around 1 in 5 health services supply them [13]. As 57% of deliveries in Nepal occurred in health facilities, an immediate postpartum IUD (PPIUD) that is inserted up to 48 h postpartum is an accessible way for postpartum women to get effective contraception [8]. The long-acting nature of the IUD makes a PPIUD an attractive option in a country like Nepal where women experience numerous barriers to accessible health care services including family planning [14].

The Government of Nepal has acknowledged the benefits of the IUD and have been promoting its use over the last decade, however, uptake has been poor. In 2016, only 1.4% of married women of reproductive age reported using an IUD with 28.2% of users discontinuing it within 12 months of insertion [8]. This low uptake may be linked to certain barriers to access. Alongside low availability of IUDs, many women experience barriers to accessing contraceptives including the IUD due to living in rural often mountainous and hilly areas with limited health facilities and providers [14, 15]. There is also a lack of knowledge on the IUD and services among women and partners which can lead to an aversion to the IUD caused by rumors and second-hand reports of side effects and a wider aversion to family planning by partners and family members [14–17].

On a provider level, a lack of trained providers in PPIUD counseling and insertion can result in poor knowledge, attitudes, and practice (KAP) towards PPIUDs. As a providers’ knowledge about IUDs influences whether they are included in family planning counseling, insufficient IUD knowledge can result in poor quality counseling and low uptake of IUDs [18–20]. Several studies assessing provider perspectives on the IUD and KAP of providers have found links between low levels of IUD knowledge and low use. Poor IUD knowledge is also related to the level of providers’ previous training [20–22]. Moreover, providers are known to have misconceptions and pre-existing bias towards LARCS including IUDs and who is eligible to use them. For example, some providers believe that IUDs are unsuitable for adolescents, women who are not married, women who have had an abortion, miscarriage, or ectopic pregnancy and postpartum women [19, 23–26]. As women’s access to IUDs depends on the providers’ ability and willingness to provide them, poor knowledge and attitudes towards IUDs can negatively impact IUD practice. Thus, denying healthy and perfectly eligible women access to IUDs, especially PPIUDs.

There is acknowledgement of the need to improve KAP among providers towards the use of IUDs through training and targeted interventions [20, 22, 25, 26]. In recent years, there has been an effort to do this in Nepal through an intervention to integrate PPFP counseling and PPIUD insertion services into routine maternity care [27]. The aim of this PPIUD intervention is to alter providers’ knowledge and behavior by training them on PPFP, including on PPIUD counseling and insertion techniques, with the ultimate aim of changing women’s knowledge and behavior through PPFP counseling and increasing the uptake of IUDs among postpartum women [27]. A study on this intervention found that PPFP counseling in the antenatal period increased by 25 percentage points with PPIUD uptake increasing on average by 4.4 percentage points [27].

Other studies in the US examining the impact IUD training interventions have on providers have found increases in KAP throughout the interventions [28, 29]. One qualitative study on this intervention in Nepal found that providers were willing to transfer their knowledge to colleagues and found no negative attitudes towards the provision of PPIUD services [30]. Another qualitative study reported an increase in PPIUD knowledge, skills, and confidence among providers [31]. While these findings are encouraging and the intervention proved to increase uptake of PPIUDs in Nepal, previous studies have not measured the PPIUD KAP of providers prior to the intervention and quantify the change throughout the intervention. This study is an analysis of the provider data from a stepped-wedge cluster randomized controlled trial of the PPIUD intervention in Nepal. Our study is novel in its aim to assess the PPIUD KAP of providers prior to the intervention and analyze whether this has changed both 6 and 24 months following the start of the intervention. The use of provider panel data followed up over 2 years is a unique feature of this study as previous studies assessing provider KAP throughout IUD interventions have a follow-up period of 1 year or less [28].

## Methods

### Parent study details

This study uses data from a broader trial analyzing the impact of an International Federation of Gynecology and Obstetrics (FIGO) led intervention to integrate PPIUD training, counseling, and provision into antenatal and delivery services in six tertiary hospitals in Nepal. The six hospitals were chosen based on high volumes of obstetric cases (> 6000 cases a year), large catchments areas and geographical location, i.e. hospitals not located in the capital city to determine if the intervention could build capacity in hospitals outside of Kathmandu valley. Using this criterion, hospitals were pair-randomized into 2 groups of 3. Baseline data collection in all hospitals began on 8 September 2015. Due to trainers travelling between hospitals to provide training, the timing of the start of the intervention by hospital in each group varied. The intervention in group 1 hospitals was implemented after three months of baseline data collection, a few weeks apart from each other in December 2015. Whereas the intervention in group 2 was implemented after nine months of baseline data collection in the summer of 2016.

### Intervention

The PPIUD intervention in Nepal was implemented by the Nepal Society of Obstetricians and Gynecologists (NESOG). The intervention itself encompasses: (1) training for healthcare providers who provide obstetric services on PPFP counseling, including PPIUD counseling and insertion; (2) providing PPFP and PPIUD information, educational and counseling materials; and (3) providing PPIUD insertion supplies to the six hospitals.

The training in the study hospitals was conducted by the Nepal Health Training Center (NHTC) of the Ministry of Health and Population (MOHP) using a national protocol. Training workshops took place over three days and covered healthy timing and spacing of pregnancies, information on different PPFP methods with an overview of PPIUDs, counseling for PPFP methods with a particular focus on PPIUDs and how to use PPFP leaflets during counseling, client assessment for PPIUD, insertion and removal of vaginal and intra-caesarean IUDs, infection prevention and management of side effects and complications, and recording and tracking of PPIUD clients.

### Sample and data collection

**Fig. 1:**
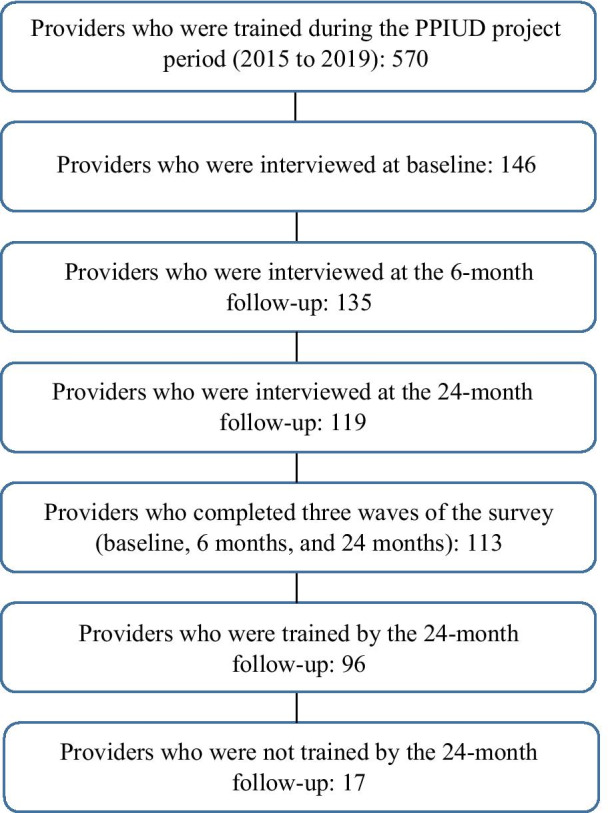
Sample of healthcare providers

A total of 570 healthcare providers were trained in PPIUD provision during the entire FIGO PPIUD project period from 2015 to 2019 [32]. Trained enumerators posted in the hospitals interviewed healthcare providers working in the Obstetrics and Gynecology departments in the study hospitals using structured questionnaires recording responses on hand-held tablets. Providers were interviewed at baseline before the FIGO intervention was implemented and were interviewed in two follow-up rounds – at 6 months, and 24 months after the start of the intervention. In total, 146 providers working in the study hospitals were recruited into the baseline survey. Of which, 135 were interviewed at 6 months and 119 providers were interviewed at 24 months. This study only includes those providers who completed all three rounds of interviews, a total of 113 providers. To better asses the KAP of providers involved in the PPIUD intervention and analyze any changes throughout the intervention we focused the main analysis on those providers who were trained at baseline or anytime in 24 months. This resulted in a final study sample of 96 trained providers, as shown in Fig. 1. The same analysis was also conducted on the 17 providers that were not trained for comparative purposes.

Though KAP survey questions were based on the Nepal National Health Facility Survey [13] and adapted for this study, many questions were purposively designed for the parent study. The parent study collected providers’ background characteristics, perspectives on contraceptive method choice, timing of initiating contraceptive use, factors considered important in making postpartum contraceptive decisions and barriers to greater use of postpartum contraceptive methods, especially PPIUD. It also covered factors that are important for expanding PPIUD services, including PPIUD knowledge, attitudes, behaviors, and practice, as well as factors important for sustainable delivery of PPIUD, such as intention to continue to provide PPIUD when moving to a new hospital.

To determine the PPIUD knowledge of providers the following four questions were used: What chance do you think that a woman using a copper IUD can get pregnant (the correct answer is ‘less than 1%’)? How long can a woman continually use the same copper IUD without removing (the correct answer is ‘12 years’)? How soon after can a woman get pregnant once her copper IUD is removed (the correct answer is ‘immediately’)? Do you think an IUD can protect against sexually transmitted infections (STIs) (the correct answer is ‘no’)?

To assess a provider’s attitude towards PPIUD we used seven questions asking if they would recommend a PPIUD to different patient populations: Would you recommend patients receive PPIUD if they were: (1) 20 years old or younger, (2) 20–29 years old, (3) 30–39 years old, and (4) 40 years old or older (positive answer is ‘yes’ for each category)? Do you recommend PPIUD to women who are not married (positive answer is ‘recommend routinely’)? How frequently do you recommend PPIUD to women who have ever had an abortion (positive answer is ‘recommend routinely’)? How frequently do you recommend PPIUD to women who have ever had an ectopic pregnancy (positive answer is ‘recommend routinely’)?

To measure a provider’s PPIUD practice three questions were used. Do you provide: (1) general counseling for family planning, (2) counseling on PPIUD, and (3) PPIUD insertion/removal (removal was included in the baseline questionnaire). The positive response for these questions is ‘yes’ for each category.

These questions were the same for interviews at the baseline, 6, and 24 months. The same questions were also used to compose three composite score indexes, one each for knowledge, attitude, and practice. Each provider was scored ‘1′ if they answered a question correctly or positively and ‘0′ if they answered incorrectly or negatively. The providers’ knowledge score ranges from 0 to 4, attitude score ranges from 0 to 7, and practice score ranges from 0 to 3, with a higher score indicating a better knowledge of, attitude towards, and practice of PPIUD.

Other questions were used to assess the changes in providers’ views on PPFP and PPIUD before and after the intervention, these include: Overall, how do you rate the IUD as compared to other methods of family planning for women in our country? How do you rate the postpartum IUD compared to other methods of family planning for immediate postpartum protection against pregnancy? (Response options were ‘worst method’, ‘worse than some’, ‘about the same’, ‘better than most’, ‘best method’ or ‘don’t know’). How important is it for women to be protected against another pregnancy during the 1 year postpartum period? (Response options were ‘not important’, ‘neutral’, ‘important’ or ‘don’t know’).

### Statistical analysis

Data were analyzed using Statistical Package for Social Sciences (SPSS) version 25 (SPSS Inc., Chicago, IL, USA). The descriptive analysis in the study uses mean and standard deviation (S.D) for continuous variables and percentage and number of participants (N) for categorical variables. Since the dataset is panel in that each provider was observed three times and the sample size is not large, we used paired sample nonparametric tests to compare differences over time periods. McNemar’s test was used to analyze any differences in categorical variables between baseline and 6-month follow-up and 6 and 24-month follow ups. KAP composite index scores are shown using mean and S.D and the Wilcoxon signed-rank test was used to assess pre-post not normally distributed continuous data between the baseline and 6-month follow-up and 6 and 24-month follow ups. All tests were 2-tailed and a P-value of less than 0.05 was considered statistically significant for all statistical analysis.

## Results

### Background characteristics

Table 1 presents the background characteristics of the 96 healthcare providers in the study. The majority of providers were female (81.3%) and were either a staff nurse, nurse, or midwife (between 62.5% at the baseline and 59.4% at 24 months). At the baseline, the providers mean age was 37.1 years and the mean years of experience in their current occupation was 11.8 years. A small number of providers had some prior training in PPIUD provision at the time of the baseline interviews (13 providers) and were retrained during the intervention, this increased to 79 providers (82.3%) by the 6-month follow-up with the remaining providers being trained by the 24-month follow-up. The mean number of hours worked per week increased through the intervention, from 41.7% of providers working over 48 h at the baseline to 46.9% at 24 months.

**Table 1:** Selected background characteristics of providers (N = 96)

Provider characteristics	Baseline (Pre-intervention) % (N)	6 months (after intervention start) % (N)	24 months (after intervention start) % (N)
Sex			
Male	18.8 (18)	18.8 (18)	18.8 (18)
Female	81.3 (78)	81.3 (78)	81.3 (78)
Mean age ± S.D. (years)	37.1 ± 8.9	38.0 ± 8.9	39.4 ± 8.9
Designation			
Obstetrician/Gynecologist	34.4 (33)	34.4 (33)	34.4 (33)
Medical Officer	1.0 (1)	1.0 (1)	1.0 (1)
Staff Nurse/Nurse/Midwife	62.5 (60)	60.4 (58)	59.4 (57)
Other	2.1 (2)	4.2 (4)	5.2 (5)
Mean year of experience in current occupation ± S.D. (years)	11.8 ± 7.9		
Received training in provision of PPIUD services			
Yes	13.5 (13)	82.3 (79)	100 (96)
No	86.5 (83)	17.7 (17)	0
Average hours working per week			
≤ 48 h	58.3 (56)	57.3 (55)	53.1 (51)
> 48 h	41.7 (40)	42.7 (41)	46.9 (45)

*PPIUD* postpartum intrauterine device, *S.D* standard deviation

### Knowledge

The PPIUD knowledge score among providers at baseline was fair with providers answering on average 2.9 questions out of 4 correctly. This increased significantly at 6 months to 3.5 (*P* < 0.001), however, dropping to 3.3 out of 4 at 24 months, though this drop is not statistically significant (Table 2). Less than half of providers at baseline (45.8%) knew that less than 1% of women have a chance of getting pregnant while using a copper IUD, increasing to 59.4% at 6 months which was not a significant increase (*P* > 0.05). At the 24-month follow-up fewer providers knew the correct answer than at 6 months (38.5%), a statistically significant drop (*P* < 0.05). Before the intervention, 78.1% of providers knew that women can continually use the same copper IUD without removing for up to 12 years. At the 6-month follow-up this increased significantly to 95.8% (*P* < 0.001) and again at 24 months to 100%. Similarly, 63.5% of providers at baseline knew that women could get pregnant immediately after removal of a copper IUD, increasing to 92.7% at 6 months (significant at *P* < 0.001) and 95.8% at 24 months. The vast majority of providers at the baseline and both follow-up interviews knew that a copper IUD cannot protect women from STIs, 99, 100 and 99% respectively with no significant changes (Table 3).

**Table 2:** Knowledge, attitude, and practice index of health care providers (N = 96)

Knowledge, attitude, and practice index	Baseline (pre-intervention)	6 months (after intervention)	24 months (after intervention)	*P*-value† (baseline to 6 months)	*P*-value† (6 months to 24 months)
Mean knowledge score ± S.D	2.9 ± 1.0	3.5 ± 0.6	3.3 ± 0.5	< 0.001***	0.080
Mean attitude score ± S.D	4.0 ± 1.2	5.3 ± 1.0	6.0 ± 1.0	< 0.001***	< 0.001***
Mean practice score ± S.D	0.9 ± 0.7	2.8 ± 0.6	3.0 ± 0.2	< 0.001***	0.002**

†Wilcoxon signed rank test

*P-value < 0.05, **P-value < 0.01, ***P-value < 0.001

*S.D* standard deviation

**Table 3:** Health care providers’ knowledge, attitudes, and practice towards family planning and PPIUD (N = 96)

Knowledge, attitudes, and practice of providers on PPIUD	Baseline (pre-intervention) % (N)	6 months (after intervention) % (N)	24 months (after intervention) % (N)	P-value‡ (baseline to 6 months)	P-value‡ (6 months to 24 months)
Knows correctly a women’s chance of getting pregnant while using copper IUD	45.8 (44)	59.4 (57)	38.5 (37)	0.055	0.014*
Knows correctly how long a woman can continually use the same copper IUD without removing	78.1 (75)	95.8 (92)	100 (96)	< 0.001***	0.125
Knows correctly how soon a woman can get pregnant after removal of copper IUD	63.5 (61)	92.7 (89)	95.8 (92)	< 0.001***	0.508
Knows correctly that IUD cannot protect women from STIs	99.0 (95)	100 (96)	99.0 (95)	1.000	1.000
Recommend PPIUD to different groups of women					
Recommend PPIUD to women less than 20 years	61.5 (59)	87.5 (84)	93.8 (90)	< 0.001***	0.146
Recommend PPIUD to women aged 20–29	99.0 (95)	100 (96)	100 (96)	1.000	–
Recommend PPIUD to women aged 30–39	97.9 (94)	100 (96)	99.0 (95)	0.500	1.000
Recommend PPIUD to women aged 40 and above	34.4 (33)	83.3 (80)	86.5 (83)	< 0.001***	0.664
Recommend PPIUD to unmarried women	17.7 (17)	41.7 (40)	77.1 (74)	< 0.001***	< 0.001***
Recommend PPIUD to women who have had an abortion	81.3 (78)	94.8 (91)	95.8 (92)	0.007**	1.000
Recommend PPIUD to women who have had an ectopic pregnancy	8.3 (8)	19.8 (19)	45.8 (44)	0.013*	< 0.001***
Practice of FP and PPIUD					
Provide general counseling for FP	70.8 (68)	99.0 (95)	99.0 (95)	< 0.001***	1.000
Provide PPIUD counseling	10.4 (10)	94.8 (91)	99.0 (95)	< 0.001***	0.219
Provide PPIUD insertion/removal	9.4 (9)	82.3 (79)	99.0 (95)	< 0.001***	< 0.001***
Providers who deemed IUD the best FP method for women after childbirth	79.2 (76)	97.9 (94)	99.0 (95)	< 0.001***	1.000
Providers who deemed IUD the best FP method for women overall	83.3 (80)	91.7 (88)	99.0 (95)	0.115	0.039*
Providers who thought it important to protect women from another pregnancy during 1 year postpartum period	33.3 (32)	31.3 (30)	34.4 (33)	0.877	0.779

‡McNemar Test

*P-value < 0.05, **P-value < 0.01, ***P-value < 0.001

*FP* family planning, *IUD* intrauterine device, *PPIUD* postpartum intrauterine device, *STIs* sexually transmitted infections

### Attitudes

Providers’ attitudes towards recommending a PPIUD to different patient populations at the baseline was middling with providers answering on average 4 out of 7 questions positively. There was a statistically significant change from the mean score of 4.0 at the baseline to 5.3 at 6-month follow-up (*P* < 0.001), and another statistically significant change between the 6 and 24-month follow ups to providers answering 6 out of 7 questions positively (*P* < 0.001) (Table 2). There was a statistically significant change in attitudes towards recommending a PPIUD to women less than 20 years old with 61.5% of providers agreeing that they would recommend a PPIUD at the baseline and 87.5% at 6 months (*P* < 0.001) with a slight increase at 24 months (93.8%). Providers had very positive views on recommending a PPIUD to women aged 20 to 29 and 30 to 39 at the baseline (99 and 97.9% recommending a PPIUD respectively) with insignificant changes between the baseline and follow ups. One third of providers at baseline would recommend a PPIUD to women aged 40 and over (34.4%). This increased significantly to 83.3% at 6 months (*P* < 0.001) with only a small insignificant increase to 86.5% from 6 to 24 months. Attitudes towards recommending a PPIUD to unmarried women was very negative before the start of the intervention with only 1 in 6 providers (17.7%) stating that they would recommend a PPIUD. At 6 months this changed significantly to 41.7% with a further significant increase to 77.1% at 24 months (*P* < 0.001). Attitude towards recommending a PPIUD to women who have had an abortion was very positive. Prior to the intervention, 81.3% of providers would recommend a PPIUD, increasing to 94.8% at 6 months (*P* < 0.01) with a small increase at 24 months (95.8%). The patient group that providers were less likely to recommend a PPIUD at baseline was women who have had an ectopic pregnancy. Only 8.3% of providers would recommend a PPIUD before the intervention, however, this increased significantly to 19.8% at the 6-month follow-up (*P* < 0.05). It increased further at 24 months with a statistically significant change (*P* < 0.001), though fewer than half of providers at 24 months (45.8%) would recommend a PPIUD to this group of women (Table 3).

### Practice

Practice of family planning and PPIUD provision was fairly low before the intervention with a mean practice score of 0.9 out of 3. By the 6-month follow-up this increased significantly to 2.8 out of 3 (*P* < 0.000) with a further significant change between 6 and 24 months to 3 out of 3 (*P* < 0.01) (Table 2). Before the intervention 70.8% of providers provided general counseling for family planning, increasing significantly to 99% at 6 months (*P* < 0.001) which remained unchanged at 24 months. The proportion of providers providing PPIUD counseling at baseline was low at 10.4%. The change between the baseline and the 6-month follow-up was statistically significant (*P* < 0.001) with 94.8% of providers providing PPIUD counseling at 6 months and 99% at 24 months. Only 9.4% of providers provided PPIUD insertion/removal before the intervention, this increased to 82.3% at 6 months and 99% at 24 months, showing statistically significant changes between baseline and 6 months and 6 and 24 months (*P* < 0.001) (Table 3).

### Postpartum pregnancy protection

Four in every five providers at the baseline deemed the IUD the best family planning method for women after childbirth (79.2%), increasing significantly to 97.9% by the 6-month follow-up (*P* < 0.001), and increasing slightly to 99% by 24 months. Additionally, 83.3% of providers before the start of the intervention deemed the IUD the best family planning method overall. Throughout the intervention this changed to 91.7% by 6 months, with a statistically significant increase to 99% between 6 and 24 months (*P* < 0.05). The proportion of providers who thought it important to protect women from another pregnancy during their 1 year postpartum period did not change significantly throughout the intervention. Only one third of providers (33.3%) thought it important before the start of the intervention, dropping slightly to 31.3% by 6 months and increasing slightly to 34.4% at 24 months (Table 3).

An analysis was also done to assess the KAP of the 17 providers who had completed the baseline interviews, and both rounds of follow-up interviews but have had no training by 24 months. The findings for this analysis can be found in Additional file 1 and Additional file 2. There was no statistically significant change in mean knowledge scores throughout the intervention with providers scoring 3.1 out of 4 before the start of the intervention (higher than those trained at 2.9) and 3.2 at 6 and 24 months (lower than those trained). The mean attitude score was lower for those not trained than those trained at the baseline and each follow-up, 3.6, 4.8, 5.4 out of 7 respectively with a statistically significant change in attitude between the baseline and 6-month follow-up (*P* < 0.01). The mean practice score prior to the start of the invention was the same for those not trained as those trained (0.9 out of 3). This did change to 1.4 at 6 months and 1.6 at 24 months (lower than those who were trained) though not a statistically significant change (see Additional file 1). There were some positive statistically significant changes between the baseline and 6-month follow-up, more notably when recommending PPIUD to women under 20 years old (P < 0.05), women who are unmarried (*P* < 0.05) and providing PPIUD counseling (*P* < 0.01), with an increase in providers providing counseling from 0 at the baseline to 58.8% at the 6-month follow-up (see Additional file 2).

## Discussion

This study has several purposes: (1) to provide a baseline of provider KAP towards PPIUDs, (2) to assess whether the PPIUD intervention in six study hospitals significantly changed provider PPIUD KAP 6 months after the start of the intervention, (3) to examine whether changes to KAP remained the same or changed further 24 months after the start of the intervention. The results of this study clearly show that the PPIUD intervention in Nepal had significant effects on providers PPIUD KAP in the study hospitals and that many of these effects were maintained over time. Similar to a study examining the impact of a provider training intervention on integration of IUDs into contraceptive care in the US [28]. Though differing somewhat from an IUD intervention in Nicaragua that found no impact on IUD uptake or on provider knowledge or attitudes after the intervention [33].

### Knowledge

Another study using baseline data to analyze the KAP of providers towards IUDs in Nepal found that general knowledge of IUD properties was ‘fair’ (answering an average of 72.5% questions correctly) [26]. This study correlates with these findings with providers having a fairly positive baseline IUD knowledge and good knowledge of how long a women can use the same copper IUD, how soon a women can conceive after removal of a copper IUD and knowing that a copper IUD cannot protect women from STIs. This contrasts with another study suggesting low levels of IUD knowledge in countries with low to moderate IUD use (much like Nepal) [21]. Providers’ knowledge also significantly increased throughout the intervention after providers had received training, evidence of a link between training and knowledge [19, 23, 28]. However, similar to a study evaluating clinicians’ knowledge and practice of the IUD in China, Kazakhstan, Laos and Mexico that found knowledge gaps with only 2.8% of respondents from all four countries (12 of 434 respondents) answering all knowledge questions correctly, there is evidence of some gaps in IUD knowledge among providers [22]. Less than half did not know that less than 1% of women have a chance of getting pregnant while using a copper IUD, with even fewer knowing this at the 24-month follow-up. This drop in providers’ knowledge between 6 and 24 months resonates with another study finding a correlation between knowledge and providers being trained up to 6 months prior to being interviewed [20].

A lack of knowledge in this area could result in providers giving postpartum women incorrect information on their chances of getting pregnant while using an IUD. This in turn could result in women refusing to use an IUD due to their misguided perception of the IUDs effectiveness. Mismatched expectations of the PPIUD caused by poor quality counseling has also been shown as a main reason for PPIUD discontinuation in Nepal [34]. Enhanced and ongoing PPIUD training throughout the intervention is needed to improve and maintain providers’ knowledge around the mechanics of the IUD and increase the quality of counseling on offer.

### Attitudes

In contrast with other studies, at the baseline providers were more positive towards recommending a PPIUD to women under 20 years old and women who have had an abortion [24, 25]. Furthermore, significantly more providers were willing to recommend a PPIUD to these groups of women after the start of the intervention. However, prior to the start of the intervention providers attitudes to recommending a PPIUD to specific patient populations was less positive, especially concerning women over 40 years old, unmarried women and women who have had an ectopic pregnancy. Therefore, resembling other studies assessing providers’ attitudes who found a reluctance to recommend IUDs to unmarried women and those with a history of ectopic pregnancy [23–26]. Encouragingly, providers’ attitudes towards recommending a PPIUD to these groups of women improved significantly throughout the intervention. This suggests some improvement in attitudes and knowledge on women’s IUD eligibility throughout the intervention [28]. Nonetheless, there is evidence of a continuing problem with provider bias towards unmarried women and more so towards women who have had an ectopic pregnancy. A noteworthy number of providers were still unwilling to recommend a PPIUD to these groups of women by the end of the study period compared to other patient populations. This could be due to continued poor knowledge of the eligibility of these women. One study in Nepal found that only 47% of providers considered unmarried women to be eligible for an IUD. The same study also found that a mere 1.2% of providers knew that women who have had an ectopic pregnancy were eligible for an IUD without screening [26]. Training for future scale ups or interventions should include information on the social and medical eligibility for a PPIUD to reduce provider bias and misunderstandings and guarantee PPIUD access to all eligible women.

### Practice

Throughout the intervention, practice of PPIUD provision among staff at the Obstetrics and Gynecology departments increased significantly from the low level at the baseline. By the 24-month follow-up, almost all the providers in the study provided general counseling for family planning, PPIUD counseling and PPIUD insertion and removal. This shows the intervention to be successful in increasing the PPIUD practice in the study hospitals and should be an incentive to run similar initiatives throughout Nepal to further increase access and uptake of PPIUDs.

### Postpartum pregnancy protection

Prior to the study, the use of IUDs for general family planning and for postpartum women was held in high regard among providers that increased further throughout the intervention. On a less positive note, prior to the start of the invention only one third of providers deemed it important to protect women from pregnancy during their first year postpartum. This did not improve throughout the intervention. As previously stated, untimely pregnancies, especially in LMICs, increases maternal and child mortality and morbidity [1–3]. In order to provide a quality PPFP and PPIUD service, providers not only need knowledge of the technicalities of an IUD, have positive attitudes towards the PPIUD and good practice of PPIUD provision, they also need to understand the importance of birth spacing and the risks that short birth to pregnancy intervals have on maternal and child health.

Improvements in PPIUD KAP were not only found among those providers who were trained in PPIUD provision, but also in those who had not been trained. Knowledge among untrained providers stayed steady throughout the intervention. However, there was a significant change in attitudes, particularly towards recommending a PPIUD to women under 20 years old and who are unmarried, and an increase in untrained providers providing PPIUD counseling between the baseline and 6 months that increased from 0 to 58.8%. This could be due to the training and intervention contributing to the initial change in KAP of trained providers, which over time and through working alongside one and other, contributed to a wider change in KAP among providers not trained, known as the spillover effect [35].

This study is subject to some limitations. As the initial study for the PPIUD intervention was not to assess the KAP of providers, questions in the survey did not follow the same structure as those asked in similar KAP studies in Nepal and, therefore, not allowing for a direct comparison [20, 26]. Related to this, the analysis in this study is limited by the questions asked in the survey. For example, the knowledge score used in this study is based on the four knowledge questions asked in the survey, whereas in similar studies up to 20 knowledge questions were asked, including questions to assess a providers’ knowledge on IUD side effects, insertion techniques, and IUD eligibility [20, 26]. Another limitation is the relatively small sample size for the analysis, especially the small sample of providers who were not trained in PPIUD provision, although this sample was not the main focus of the study. This study only provided a descriptive analysis of changes in providers’ KAP. More analysis to investigate factors that drive the changes is needed. The advantage of this study is to use panel data and have three waves of measurement for the same participants. Comparisons within each provider control for time-invariant confounders. Thus, an intervention effect can be attributable to the significant difference in KAP for the same providers at baseline and 6 months after the start of the intervention. However, reasons need to be investigated behind the significant differences in KAP between providers at 6 months and 24 months after the start of the intervention. The questionnaire did not contain a sufficient number of questions about providers’ changing characteristics to support such an investigation. As there is more than a 1 year time gap between the 6-month survey and the 24-month survey. Significant differences in KAP between providers at 6 and 24 months could be related to the time providers have been practicing PPIUD provision after their initial training. Many other conditions, such as changing facility environment, may also contribute to providers’ KAP changes, which is also not captured in the provider survey. Finally, the results are based on responses of providers and answers to questions on attitudes or practice may be prone to misreporting or to “courtesy” bias.

Nonetheless, this study is the first to examine baseline KAP of a panel of PPIUD providers in Nepal using data captured during a PPIUD intervention to assess any changes in provider KAP throughout. By using 24-month follow-up data (the typical follow-up period for similar studies is 1 year or less), this study is particularly unique not only adding to the literature in Nepal but to the wider literature on the KAP of providers of PPIUDs worldwide. Our evaluation demonstrates that the KAP of the providers in the study hospitals changed significantly throughout the course of the PPIUD intervention, having a reasonable impact on providers’ PPIUD knowledge, their attitudes towards recommending a PPIUD to certain groups of women, and on PPIUD practice.

## Conclusion

The PPIUD intervention had a positive effect on the KAP of providers in the study hospitals yet its effectiveness may be improved through ongoing and more in-depth training. The intervention had a fairly positive effect on providers’ knowledge, though highlighted a problem with providers maintaining their knowledge on a women’s chance of getting pregnant while using a copper IUD. The intervention showed an encouraging effect on providers’ attitudes, with an increase in providers recommending a PPIUD to women aged 40 years and older, unmarried women, and women who have had an ectopic pregnancy. Yet, there is room for improvement to encourage even more providers to recommend a PPIUD to these groups of women. Although PPIUD practice among providers increased substantially throughout the intervention, providers’ views on the importance of protecting women from pregnancy during their 1 year postpartum period was poor and did not improve throughout the intervention. The study results suggest that the interventions impact on the KAP of providers could be improved further by: (1) providing ongoing refresher training to ensure that providers increase and maintain their knowledge on the PPIUD, (2) enhancing training to include social and medical PPIUD eligibility, especially of unmarried women and women who have previously had an ectopic pregnancy to ensure that these group of women are not denied a PPIUD, (3) ensuring that providers are aware of the negative consequences of denying PPIUD to eligible groups of women to reduce provider bias towards certain patient populations, and (4) placing more emphasis during training on the importance of birth spacing and protecting women from pregnancy during their first year, and ideally their second year, postpartum period.

## Supplementary Information

**Additional file 1.** Knowledge, attitude, and practice index of health care providers not PPIUD trained (N = 17).

**Additional file 2. **Health care providers’ knowledge, attitudes, and practice towards family planning and PPIUD among those not PPIUD trained (N = 17).

## Data Availability

De-identified dataset used and/or analyzed during the current study will be available from the second author on reasonable request.

## Abbreviations

ANC: Antenatal care
CREHPA: Centre for Research on Environment Health and Population Activities
FIGO: International Federation of Obstetricians Gynaecologists
IUC: Intrauterine contraception
IUD: Intrauterine device
KAP: Knowledge, attitude, and practice
LMICs: Low-and middle-income countries
MOHP: Ministry of Health and Population
NESOG: Nepal Society of Obstetricians and Gynaecologists
NHTC: Nepal Health Training Centre
PPFP: Postpartum Family Planning
PPIUD: Postpartum intrauterine device
