# Supplementary material for: Assessing knowledge, attitudes, and practice of health providers towards the provision of postpartum intrauterine devices in Nepal: a two-year follow-up
Source: Reprod Health. 2021 Feb 17;18:43. doi: 10.1186/s12978-021-01099-7 (PMC7891136; doi:10.1186/s12978-021-01099-7)
Supplement: Supplementary file 1 — Additional file 1. Knowledge, attitude, and practice index of health care providers not PPIUD trained (N = 17). [file 12978_2021_1099_MOESM1_ESM.docx]

**Additional file 1**

**Table 4: Knowledge, attitude, and practice index of health care providers not PPIUD trained (N=17)**

| **Knowledge, attitude, and practice index** | **Baseline (pre-intervention)** | **6 months (after intervention)** | **24 months (after intervention)** | **P-value† (baseline to 6 months)** | **P-value† (6 months to 24 months)** |
| --- | --- | --- | --- | --- | --- |
| **Mean knowledge score ± S.D** | 3.1 ± 0.8 | 3.2 ± 0.9 | 3.2 ± 0.6 | 0.414 | 0.813 |
| **Mean attitude score ± S.D** | 3.6 ± 0.9 | 4.8 ± 1.4 | 5.4 ± 1.7 | 0.005** | 0.440 |
| **Mean practice score ± S.D** | 0.9 ± 0.3 | 1.4 ± 0.9 | 1.6 ± 0.9 | 0.059 | 0.273 |

†Wilcoxon signed rank test

*P-value <0.05, **P-value <0.01, ***P-value <0.001

Abbreviations: S.D, standard deviation.
