# Supplementary material for: Assessing knowledge, attitudes, and practice of health providers towards the provision of postpartum intrauterine devices in Nepal: a two-year follow-up
Source: Reprod Health. 2021 Feb 17;18:43. doi: 10.1186/s12978-021-01099-7 (PMC7891136; doi:10.1186/s12978-021-01099-7)
Supplement: Supplementary file 2 — Additional file 2. Health care providers’ knowledge, attitudes, and practice towards family planning and PPIUD among those not PPIUD trained (N = 17). [file 12978_2021_1099_MOESM2_ESM.docx]

**Additional file 2**

**Table 5: Health care providers’ knowledge, attitudes, and practice towards family planning and PPIUD among those not PPIUD trained (N=17)**

| **Knowledge, attitudes, and practice of providers on PPIUD** | **Baseline (pre-intervention)**  **% (N)** | **6 months (after intervention)**  **% (N)** | **24 months**  **(after intervention)**  **% (N)** | **P-value‡ (baseline to 6 months)**  **% (N)** | **P-value‡ (6 months to 24 months**  **% (N)** |
| --- | --- | --- | --- | --- | --- |
| Knows correctly a women’s chance of getting pregnant while using copper IUD  Knows correctly how long a woman can continually use the same copper IUD without removing  Knows correctly how soon a woman can get pregnant after removal of copper IUD  Knows correctly that IUD cannot protect women from STIs | 58.8 (10)  70.6 (12)  82.4 (14)  100 (17) | 58.8 (10)  94.1 (16)  70.6 (12)  100 (17) | 41.2 (7)  88.2 (15)  88.2 (15)  100 (17) | 1.000  0.125  0.500  -- | 0.508  1.000  0.375  -- |
| **Recommend PPIUD to different groups of women**  Recommend PPIUD to women less than 20 years  Recommend PPIUD to women aged 20-29  Recommend PPIUD to women aged 30-39  Recommend PPIUD to women aged 40 and above  Recommend PPIUD to unmarried women  Recommend PPIUD to women who have had an abortion  Recommend PPIUD to women who have had an ectopic pregnancy | 29.4 (5)  100 (17)  100 (17)  35.3 (6)  5.9 (1)  88.2 (15)  0 | 70.6 (12)  100 (17)  100 (17)  58.8 (10)  41.2 (7)  94.1 (16)  17.6 (3) | 82.4 (14)  88.2 (15)  100 (17)  76.5 (13)  52.9 (9)  88.2 (15)  47.1 (8) | 0.016*  --  --  0.289  0.031*  1.000  0.250 | 0.625  0.500  --  0.453  0.687  1.000  0.063 |
| **Practice of FP and PPIUD**  Provide general counselling for FP  Provide PPIUD counselling  Provide PPIUD insertion/removal | 88.2 (15)  0  0 | 76.5 (13)  58.8 (10)  0 | 88.2 (15)  64.7 (11)  11.8 (2) | 0.687  0.002**  -- | 0.625  1.000  0.500 |
| Providers who deemed IUD the best FP method for women after childbirth  Providers who deemed IUD the best FP method for women overall | 64.7 (11)  76.5 (13) | 94.1 (16)  94.1 (16) | 94.1 (16)  94.1 (16) | 0.125  0.375 | 1.000  1.000 |
| Providers who thought it important to protect women from another pregnancy during 1 year postpartum period | 23.5 (4) | 17.6 (3) | 17.6 (3) | 1.000 | 1.000 |

‡McNemar Test

*P-value <0.05, **P-value <0.01, ***P-value <0.001

Abbreviations: FP, family planning; IUD, intrauterine device; PPIUD, postpartum intrauterine device; STIs, sexually transmitted infections.
